# The comparison of endoscopic and open saphenous vein harvesting techniques in terms of the complications to the lower limb

**DOI:** 10.1186/1749-8090-8-S1-O197

**Published:** 2013-09-11

**Authors:** W Piotrowicz, M Kempa, P Kwinecki, M Piorkowski, R Cichon

**Affiliations:** 1 Lower Silesian Heart Diseases Centre MEDINET, Wroclaw, Poland

## Background

The greater saphenous vein is commonly used in coronary revascularization. Beside classical methods of vein harvesting, endoscopic technique is applied. The introduction of endoscopic vein harvesting remains still controversial and therefore needs some more data resulting from application of this method. This study was performed to investigate the influence of endoscopic and open saphenous vein harvesting techniques on short- and mid-term effects on the lower limb after coronary artery bypass grafting.

## Methods

The data has been collected on isolated CABG/OPCAB procedures with the use of saphenous vein graft carried out between April and December 2010 in one center. The population consisted of 216 patients (156 male, 60 female) aged 39-87 (average 69,3). 41 patients underwent endoscopic vein harvesting (EVH) and in 175 patients the vein was harvested with the open method (open vein harvesting - OVH). The assessment included all basic patients’ data, procedure data, postoperative observation data and a telephone survey 3 years after the procedure consisting of questions about eventual lower limb problems.

## Results

Early complications (up to 14 days after the procedure) to the lower limb included hematomas – 4,9% in the EVH group, 2,3% in the OVH group (p>0,1), bloody/serous leakage – 2,4% in the EVH group, 7,4% in the OVH group (p>0,1) and inflammation – 1,7%, only in the OVH group (p>0,1). Late complications (within 3 years after the procedure) included edemas – 10,3% in the EVH group, 26,6% in the OVH group (p<0,05), pain – 5,1% in the EVH group, 20,7% in the OVH group (p<0,05) and sensory disorders – 7,7% in the EVH group, 16,6% in the OVH group (p>0,1). Other complications – prolonged healing (2,4%), inflammation (2,4%), infection (1,8%) and lividness (1,2%) appeared only in the OVH group (p>0,1).

## Conclusion

The study proved that endoscopic saphenous vein harvesting technique reduces short- and mid-term lower limb wound complications.

